# Insights of Phenolic Pathway in Fruits: Transcriptional and Metabolic Profiling in Apricot (*Prunus armeniaca*)

**DOI:** 10.3390/ijms22073411

**Published:** 2021-03-26

**Authors:** Helena Gómez-Martínez, Francisco Gil-Muñoz, Almudena Bermejo, Elena Zuriaga, Maria L. Badenes

**Affiliations:** Instituto Valenciano de Investigaciones Agrarias (IVIA), CV 315, Km 10,5, 46113 Moncada, Valencia, Spain; gomez_hel@gva.es (H.G.-M.); gil_framuna@externos.gva.es (F.G.-M.); bermejo_alm@gva.es (A.B.); garcia_zur@gva.es (E.Z.)

**Keywords:** phenolic pathway, FLS, DFR, PAL, fruits

## Abstract

There is an increasing interest in polyphenols, plant secondary metabolites, in terms of fruit quality and diet, mainly due to their antioxidant effect. However, the identification of key gene enzymes and their roles in the phenylpropanoid pathway in temperate fruits species remains uncertain. Apricot (*Prunus armeniaca*) is a Mediterranean fruit with high diversity and fruit quality properties, being an excellent source of polyphenol compounds. For a better understanding of the phenolic pathway in these fruits, we selected a set of accessions with genetic-based differences in phenolic compounds accumulation. HPLC analysis of the main phenolic compounds and transcriptional analysis of the genes involved in key steps of the polyphenol network were carried out. Phenylalanine ammonia-lyase (PAL), dihydroflavonol-4-reductase (DFR) and flavonol synthase (FLS) were the key enzymes selected. Orthologous of the genes involved in transcription of these enzymes were identified in apricot: *ParPAL1*, *ParPAL2*, *ParDFR*, *ParFLS1* and *ParFLS2*. Transcriptional data of the genes involved in those critical points and their relationships with the polyphenol compounds were analyzed. Higher expression of *ParDFR* and *ParPAL2* has been associated with red-blushed accessions. Differences in expression between paralogues could be related to the presence of a BOXCOREDCPAL cis-acting element related to the genes involved in anthocyanin synthesis *ParFLS2*, *ParDFR* and *ParPAL2*.

## 1. Introduction

Apricot (*Prunus armeniaca*) is an important fruit crop in Mediterranean basin countries and Asia, with a wide diversity in pomological characteristics and fruit quality properties due to its different diversification centers [1]. Apricots are a good source of vitamins, carotenoids, and polyphenols [2], which makes this species a good choice from a nutraceutical point of view [3].

Higher plants have several defense mechanisms against biotic and abiotic stresses. Some of these mechanisms result in the synthesis of a large number of secondary metabolites. Flavonoids are one of these defense-related secondary metabolites, being a family of polyphenols synthesized by the phenylpropanoid biosynthetic pathway [4]. These secondary metabolites remain in different plant organs and accumulate on the plant surface [5]. In the case of flavonoid compounds, their accumulation is unequally distributed within tissues, as its concentration is higher in the peel of several fruits such as apple [6], peach [7], or apricot [8].

Polyphenols have been identified as secondary metabolites with great antioxidant activity [9,10,11]. In recent years, there is an increasing interest in them as contributors to fruit quality and dietary properties. In the case of apricot, the fruit peel is an excellent source of phenolic compounds. The main phenylpropanoid-derivate secondary metabolites in apricot are chlorogenic and neochlorogenic acids, two caffeate derivates monolignols, while the main flavonols are rutin and quercetin-3-glucuronide [12].

**Figure 1:**
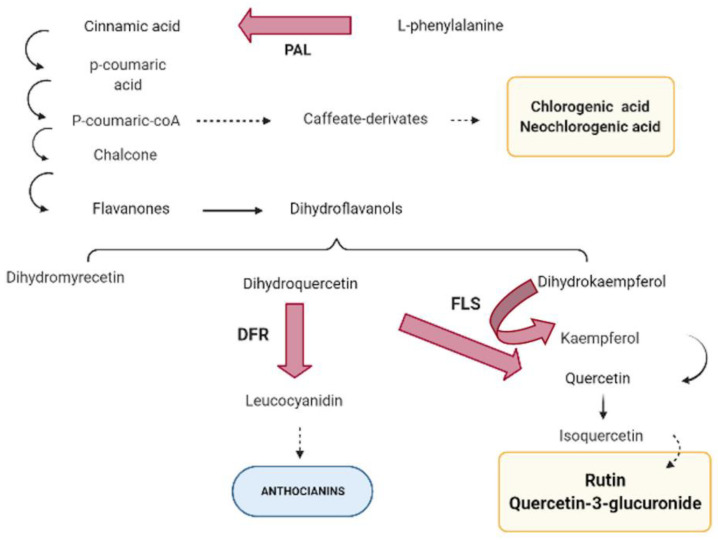
Phenolic biosynthesis pathway. Red arrows represent the key genes studied: PAL (phenylalanine ammonia-lyase), FLS (flavonol synthase), and DFR (dihydroflavonol-4-reductase). Yellow squares represent the main metabolites contributing to apricot antioxidant capacity.

Phenylpropanoid biosynthesis starts from the conversion of L-phenylalanine into cinnamic acid due to the action of phenylalanine ammonia-lyase (PAL) (Figure 1).

Phenylalanine ammonia-lyase (PAL) has been described as the first enzyme in the phenylpropanoid pathway, considered a key regulatory point between primary and secondary metabolism through conversion of L-phenylalanine into cinnamic acid [13]. PAL is encoded by a multi-gene family, in which the number of genes involved depends on the species. In *Arabidopsis* and *Nicotiana*, four PAL-encoding genes have been described [14,15,16], five in poplar [17], and two in different *Prunus* species [18]. In the following step, cinnamic acid 4-hydroxylase converts cinnamic acid into 4-coumaric acid, to which a coenzyme-A is added due to the action of 4-coumarate-CoA ligase, giving 4-coumaroyl-CoA as a result. At this point, the pathway can branch off to the caffeate derivates biosynthesis, producing chlorogenic and neochlorogenic acids. Alternatively, 4-coumaroyl-CoA is also used by chalcone synthase to catalyze the synthesis of chalcone, which is isomerized to colorless flavanones. These compounds can be hydroxylated at three different positions, by three different flavonoid hydroxylases, producing a group of dihydroflavonols. Then, the phenolic pathway can branch off to the flavonols biosynthesis due to the action of flavonol synthase (FLS). This enzyme uses dihydroflavonols (dihydroquercetin, dihydrokaempferol, or dihydromyricetin) as a substrate to produce kaempferol, quercetin, or myricetin, the main precursors of some flavonols such as rutin or quercetin-3-glucuronide. Previous works have identified FLS-encoding genes in Arabidopsis [19,20]. In addition, FLS has been related with dihydroflavonols catalysis to flavonol but also it has been related to anthocyanin accumulation [20,21]. On the other hand, dihydroflavonol-4-reductase (DFR) enzyme controls one of the limiting steps of the anthocyanin pathway, reducing dihydroflavonols to leucoanthocyanidins [22,23,24], therefore using the same substrate as FLS. Several DFR-encoding genes have been identified in different species [23,25,26,27]. Although phenolic metabolism regulation remains ambiguous in some points, various studies have identified the role of MYB transcription factors in phenolic synthesis regulation [28,29,30].

Nevertheless, although the main steps of the metabolic pathway are described, the identification of key gene enzymes and their roles in the phenylpropanoid pathway of some fruit crops remain uncertain. As the first step for a better understanding of the phenolic pathway in fruits, we selected a set of apricot accessions from the IVIA’s apricot breeding program with genetic-based differences in phenolic compound accumulation [8].

Fruit phenolic content of the genotypes selected was evaluated and compared with the genetic expression of genes encoding key enzymes of the phenolic biosynthesis pathway related to primary phenolic compounds (PAL), anthocyanin biosynthesis (DFR), and secondary phenolic metabolites (FLS). Since FLS and DFR use the same substrate for producing either flavonols or anthocyanins, respectively, their possible role in flavonol accumulation in apricot should be studied. Characterization of the expression of main genes acting in the phenolic pathway and its relationship with fruit polyphenol content will provide tools to unravel the phenolic pathway of fruit species. This information will be of interest in breeding programs aimed at increasing fruit quality and useful for the promotion of fruit consumption.

## 2. Results

### 2.1. Apricot Polyphenol Content

Total polyphenol content and the main phenolic compounds were evaluated for each year of study, including the two-years average content. Results are indicated in Table 1 and Table S1. Significant differences were found among all genotypes studied. The higher values were obtained in genotypes with an important red-blush color on the skin: ‘Dama Rosa’, ‘GG9310’, ‘GG979’, ‘GP9817’, and ‘HM964’.

**Figure 2:**
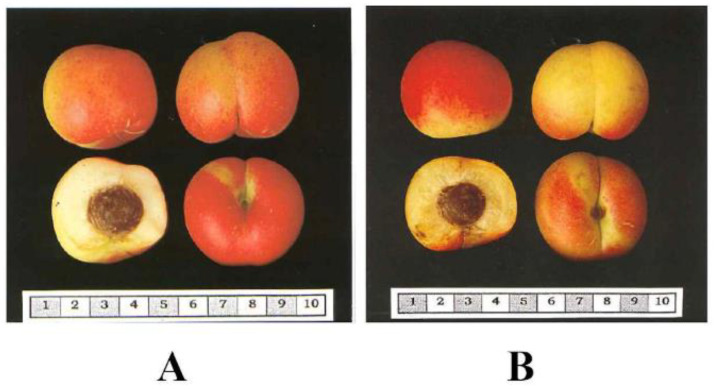
Examples of apricot fruits from Mediterranean varieties used as genitors in the breeding program with high red-blush on the skin. This trait is related to anthocyanin content. (**A**). Fruits from ‘Ginesta’ (**B**). Fruits from ‘Palau’.

The most important disease affecting *Prunus* species is caused by the Plum Pox Virus (PPV). The donor of PPV resistance ‘Goldrich’ and hybrids between ‘Goldrich’ and the Mediterranean autochthonous varieties (Ginesta and Palau) (Figure 2), presented more than 50% of red-blush in the skin and the highest amounts of total polyphenol content. The variety ‘Mitger’ contributes as well to the total polyphenol content of hybrids. Results indicated that hybrids from these three varieties (Ginesta, Palau and Mitger) crossed with ‘Goldrich’ produced genotypes with interesting polyphenol content.

**Figure 3:**
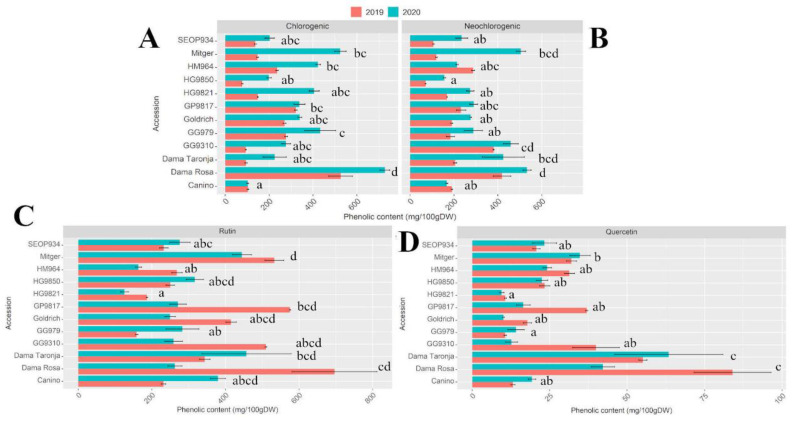
Chlorogenic (**A**), neochlorogenic (**B**), rutin (**C**) and quercetin-3-glucuronide (**D**) contents (mg/100 g DW) in 2019 (red) and 2020 (blue). Values represent the mean of 3 biological replicates, bars represent standard deviation. Different letters represent statistical differences between genotypes.

The main secondary phenolic compounds: rutin, quercetin, chlorogenic, and neochlorogenic acid were analyzed and a similar trend was obtained. ‘Dama Rosa’ showed the highest concentrations for all the studied compounds. ‘Goldrich’ hybrids ‘Dama Rosa’, ‘Dama Taronja’, and ‘GP9817’ showed higher content of neochlorogenic acid and rutin compared to the other accessions (Figure 3). Differences among cultivars were found in both years (Table S1).

### 2.2. Putative Orthologous and Phylogenetic Analysis

BLAST analysis using *P. persica* and *A. thaliana* DFR, FLS, and PAL identified a total of five genes in P. armeniaca: *ParDFR* (PARG07267), *ParFLS1* (PARG08425), *ParFLS2* (PARG08426), *ParPAL1* (PARG18722), *ParPAL2* (PARG02214). Table S2 shows high (>95%) conservation between peach and apricot for all genes. PAL genes were located in different linkage groups in both species, and as a consequence, in different synteny blocks. *PpePAL1* was located in LG2, meanwhile apricot was located in LG5. However, *PpePAL2*, located in LG6, matched in LG1 in apricot. *PpeDFR*, *PpeFLS1*, and *PpeFLS2* were located in LG1 in peach, but they match with LG2 in *Prunus armeniaca*. All the predicted locations matched with the synteny between these regions in apricot and peach. In addition, *Arabidopsis thaliana* and *Prunus armeniaca* also had a high identity (>80%) for PAL, more than 70% for *ParDFR* and 60% for *ParFLS1* and 45.65% for *ParFLS2* (Table S3). In addition, protein alignment revealed a high conservation among *Prunus* and *Arabidopsis thaliana* (Tables S4 and S5). *ParPAL1* and *ParPAL2* showed around 80% of similarity with *AtPAL1* and *AtPAL2*, respectively. Regarding DFR, similarity was around 70% mean. FLS showed the lowest similarity with 57% and 43% for FLS1 and FLS2. A similar trend was observed for *Prunus persica* and *Arabidopsis thaliana*.

**Figure 4:**
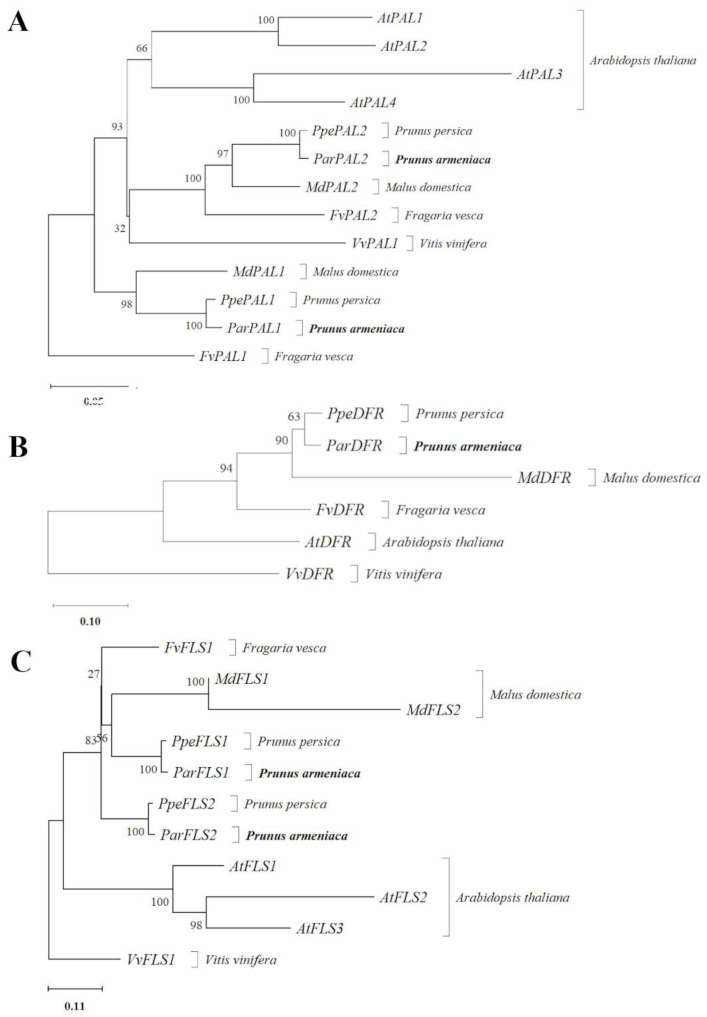
Neighbor-Joining phylogenetic tree for the proteins encoded by PAL (**A**), DFR (**B**), and FSL (**C**) genes. Each tree was bootstrapped 1000 times. Numbers close to each branch represent the percentage of replicate trees in which the associated taxa clustered together in the bootstrap test. Trees are drawn to scale according to evolutionary distances (*p*-distance), included under each tree representing the number of substitutions per site.

*ParPAL1* and the putative *PAL1* orthologous from *Prunus persica* and *Malus domestica* were clustered together. *ParPAL2* and its putative orthologous were grouped in a different cluster which showed the differences among both paralogs. The phylogenetic tree of phenylalanine ammonia-lyase proteins (Figure 4A), showed that all *Arabidopsis thaliana* proteins clustered together.

The phylogenetic tree revealed that DFR proteins of *Prunus persica* and *Prunus armeniaca* clustered together, being closed to its orthologous from *Malus domestica* (Figure 4B).

The predicted proteins encoded by FLS genes of *Arabidopsis thaliana* grouped in a cluster. On the other hand, *Prunus persica* predicted proteins from *PpeFLS2* and *ParFLS2* were grouped in the same cluster, as were *Prunus armeniaca PpeFLS1* and *ParFLS1*. However, *Fragaria vesca* predicted sequences encoded by *FvFLS* clustered in another tree branch with the *Malus domestica* proteins group (Figure 4C).

### 2.3. Gene Expression

**Figure 5:**
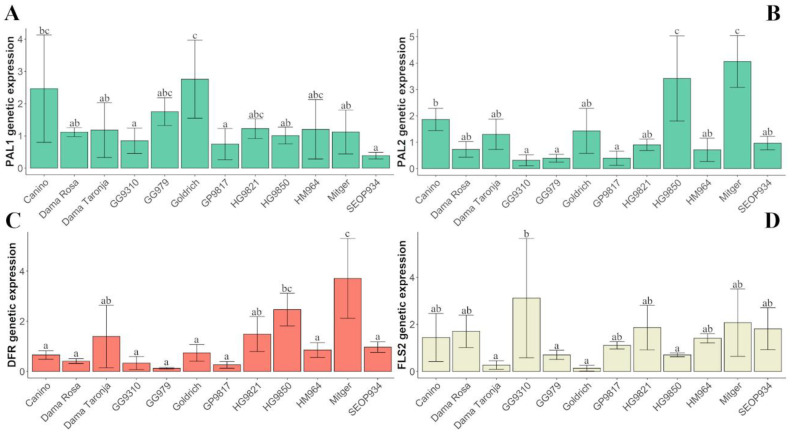
Genetic expression (average of both years of study) of *ParPAL1* (**A**), *ParPAL2* (**B**), *ParDFR* (**C**), and *ParFLS2* (**D**). Bars represent standard deviation. Different letters represent statistically significant differences.

Genetic expression of the genes studied (*ParPAL1*, *ParPAL2, ParDFR*, *ParFLS1*, *ParFLS2)* did not show a year effect but a genotype effect (Kruskal-Wallis test). Subsequently, we found minor differences in gene expression among genotypes (Figure 5, Table S6).

Genetic expression of *ParPAL1, ParPAL2, ParDFR,* and *ParFLS2* showed significant differences among genotypes (Figure 5). Concerning the expression of flavonol-synthase encoding gene *ParFLS1*, no significant differences among genotypes were observed.

Regarding the expression of phenylalanine ammonia-lyase (*ParPAL1* and *ParPAL2*), only the variety ‘Goldrich’ showed significant differences on *PAL1* and two genotypes showed significant differences on *PAL2* (‘Mitger’ and HG9850).

### 2.4. Contribution of ‘Goldrich’ to Phenolic Compounds Content and Genetic Expression

In this study, ‘Goldrich’ used as donor of resistance to PPV in most apricot breeding programs worldwide and the main contributor to the hybrids included in this study, was evaluated as contributor of compounds for fruit quality (Table 2).

The variety ‘Goldrich’ showed a significant genetic effect on total polyphenol content. A coefficient of 382.28 mg 100 g^−1^ DW, which represents more than 45% of the general average of the population. A similar genetic effect was observed for the specific phenolic compounds, except quercetin-3-glucuronide, in which the genetic effect of ‘Goldrich’ was not significant. The genetic effect of ‘Goldrich’ for neochlorogenic and chlorogenic acids were 127.94 and 135.22 mg 100g^−1^, representing 56% and 57% of the general average, respectively. For rutin, the coefficient was 110.7 mg 100g^−1^ (37.3% of the general average).

Concerning genetic expression, the cultivar ‘Goldrich’ had a genetic effect on the expression of all the genes studied. This effect was significant for the five genes studied *ParPAL1*, *ParPAL2 ParDFR*, *ParFLS1*, *ParFLS2*, (Table 3). The genetic effect of ‘Goldrich’ varies from 58.2% in *ParFLS2* to 98.7% in *ParDFR*.

### 2.5. Relationships between Gene Expression and Phenolic Compound Accumulation

A correlation analysis performed among compounds and expression of genes studied revealed a significant correlation between neochlorogenic acid and the rest of the phenolic compounds. (Table 4).

*ParDFR* expression revealed a positive correlation with *ParAL2* (0.8) but also showed positive correlation with *ParFLS1*, which also correlated positively with *ParPAL2*. The gene expression obtained indicates interaction among the genes selected in key steps of the polyphenol pathway.

**Figure 6:**
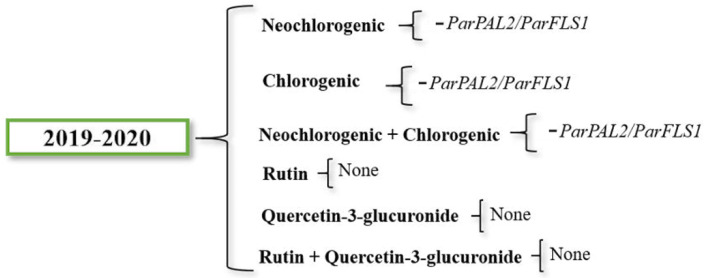
Significant effects of gene expression data from the linear regression model contributing to the content of each compound in both years studied. Negative symbol means negative contribution.

To complete the previous study, we studied the relationships between the gene expression and each phenolic compound content through a linear regression model (Tables S7 and S8). Ratios such as *PAL*/*FLS*, *PAL*/*DFR* or *FLS*/*DFR* were analyzed in order to study the differences in gene expression balance and its possible relationship with a preferential biosynthesis of anthocyanins, flavonols or caffeate-derivates. The trend between the phenolic compounds content and the expression of genes obtained is summarized in Figure 6.

Both neochlorogenic and chlorogenic acid content were negatively influenced by *ParPAL2*/*ParFLS2* ratio. Due to neochlorogenic and chlorogenic acids being synthetized in the same pathway branch, the correlation between their content and the gene expression was also evaluated together. Data from the two-years average revealed a negative impact of *ParPAL2*/*ParFLS1* in the neochlorogenic and chlorogenic total content. Concerning rutin and quercetin-3-glucuronide content, no significant correlation was found. The gene expression effect on the levels of accumulation of all the compounds was low.

### 2.6. Cis-Acting Elements Analysis

**Figure 7:**
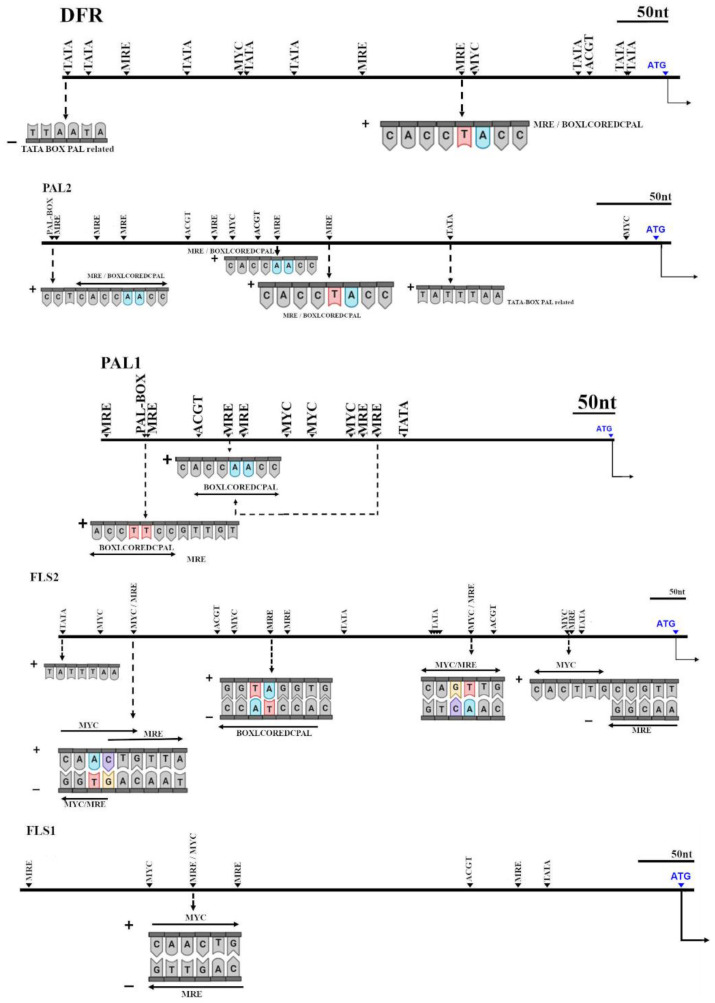
Analysis of cis-acting elements on 1500 bp upstream from start codon (ATG) sequences of *Prunus armeniaca DFR*, *PAL*, and *FLS* genes. MRE: MYB-like Recognition Element; MYC: MYC-like recognition sequence; TATA: TATA box-like; BOXLCOREDCPAL: Consensus of the putative “core” sequences of box-L-like PAL promoter region.

Due to the correlation among expression of some genes, a study of upstream sequences to find cis-acting elements recognized by *MYB-like* transcription factors was carried out (Figure 7).

In *ParDFR*, we found at 694 bp upstream from ATG, a *TATA-BOX-PAL* related, next to other *TATA-box-like* motif and *MRE* (a *MYB*-recognition element). In addition, a *MYC* motif was found together with a *TATA-box-like*. Furthermore, at 238 bp upstream from ATG, a *MRE* was found encoding also a *BOXLCOREDCPAL*, a motif related with the *PAL* promoter region. This *MRE* was closed to a *MYC* motif.

In *ParPAL2*, 403 bp and 255 bp upstream from ATG we found an *MRE* encoding a *BOXLCOREDCPAL* with a different sequence from the one found in *ParDFR*. However, 220 bp upstream from ATG we found the same *MRE* encoding a BOXLCOREDCPAL as found in DFR. In addition, a *TATA*-*BOX*-*PAL* related was found 139 bp upstream.

However, in *ParPAL1* we did not find the same *MRE* encoding the *BOXLCOREDCPAL*, found in *ParDRF* and PAL2 upstream. Indeed, we found 551 bp upstream from ATG, also the same *MRE* motif but differing only in a nucleotide. On the other hand, in 276 bp upstream we found an *MRE* encoding a *PAL-box-like* motif, identical as found twice in PAL2.

In *ParFLS1*, we found four *MRE*, but none of them encoded a *PAL-box-like* motif. However, 438 bp upstream from ATG, we found a *MYC* motif, but also an MRE antisense.

In *ParFLS2*, we found 572 bp upstream the same MRE encoding a *BOXLCOREDCPAL*, as found in *ParDFR* and *ParPAL2*. Furthermore, 765 bp upstream we found the same *MYC/MRE* motif found in *ParFLS1*. Moreover, the same cis-acting element was found antisense 289 bp upstream from ATG, but antisense.

## 3. Discussion

### 3.1. Polyphenol Content

The total polyphenol and individual phenolic compounds analyzed were genotype-dependent. The higher values corresponded to genotypes derived from varieties characterized by important red skin color, such as the Mediterranean autochthonous varieties ‘Ginesta’, ‘Palau’, and ‘Mitger’ or the donor of resistance to PPV ‘Goldrich’. This fact agrees with the references in which polyphenol content, anthocyanins and red color of fruits are related [31,32]. On the other hand, the linear model indicates that contribution of the variety ‘Goldrich’ to the content of polyphenols is remarkable in agreement with previous results [8]. This suggests that the introgression of resistance to PPV (the most important objective of the apricot breeding programs worldwide) is not negatively affecting the fruit quality of apricot, another important objective of the apricot breeding programs from the Mediterranean basin.

Genetic expression of *ParPAL1* was the highest in ‘Goldrich’. This accession has been previously identified as a contributor of phenolic compounds content in its derived hybrids [8]. Indeed, phenylalanine ammonia-lyase (PAL) plays a significant role in the phenylpropanoid metabolism pathway. PAL, as the first key enzyme in phenylpropanoid biosynthesis, catalyzes the conversion of L-phenylalanine to cinnamic acid, linking primary metabolism with secondary metabolism and becoming a speed-limiting step in phenylpropanoid metabolism [33]. In *Prunus* species, this genetic family consists of two *PAL* members [18] and in our study they were identified in apricot by synteny with peach (*ParPAL1* and *ParPAL2*). We have identified the ‘Goldrich’ genetic effect in increasing *ParPAL1* expression. This result, along with the previously described effect in the increase of phenolic compounds [8], suggests that this gene contributes to phenolic accumulation in the group of genotypes studied.

The next critical step analyzed is the one where the phenolic pathway branches off towards anthocyanins or flavonol synthesis. Dihydroflavonol reductase (DFR) is an enzyme that catalyzes the reduction from dihydroflavonols to anthocyanins biosynthesis [22,23,24]. Our results revealed major *ParDFR* expression in hybrids from cultivars with high percentages of red-blush [34]. This red coloration could be associated with anthocyanin accumulation as shown by previous studies in apricot [35]. Consequently, our results may suggest a higher *ParDFR* expression in those cultivars with high percentages of red-blush on the fruit skin.

Alternatively, flavonol synthase (FLS) catalyzes the reaction from dihydroflavonols to flavanols, a group of flavonoids in which rutin and quercetin-3-glucuronide are found. In apricot, two *FLS* encoding genes are present: *ParFLS1* and *ParFLS2*. A two crop years average revealed lower expression of *ParFLS2* in those genotypes without contribution of autochthonous genitors characterized by red-blush fruits. High expression was obtained in hybrids from cultivars with an important percentage (>50%) of fruit skin covered by a red-blush with a high intensity of over color [34]. Additionally, most of the cultivars of this group were also reported as the accessions with major total content in polyphenols. These results are in agreement with previous works, indicating that expression of *FLS* could be related to phenolic biosynthesis and also linked with anthocyanins accumulation [20,21].

At gene expression level, the ‘Goldrich’ effect was correlated positively with *ParPAL1*. Taking into account that ‘Goldrich’ has a positive contribution on polyphenol content, this fact suggests that *ParPAL1* expression levels are related to the accumulation of phenolic compounds. On the other hand, we have not found correlations between individual *ParPAL1* gene expression and any studied compound (Table 4). This fact can be explained because the analysis was carried out at full maturity, whereas main polyphenol compounds biosynthesis might occur in previous fruit stages. As neochlorogenic and chlorogenic content were influenced negatively with *ParPAL2*/*ParFLS1* ratio (Figure 6), we suggest that *ParPAL2* could be unbalancing the pathway to anthocyanin biosynthesis, having a negative impact on the synthesis of these compounds.

### 3.2. Genes and Its Inference in Polyphenols Pathway

Previous studies related *MYB* transcription factors with phenolic biosynthesis in various species [29,30]. In fact, Hartmann et al. [28] showed a relation between cis-acting elements recognized by *R2R3-MYB* (or MYB-recognition element (*MRE*)), *BZIP* (ACGT-element), and *BHLH* (CANNTG motif) with phenylpropanoid biosynthesis genes.

Both *PAL* and *FLS* putative orthologous analysis resulted in two genes per enzyme identified in the *P. armeniaca* genome. Genome duplication is common among plants, leading to the duplication of genes [36]. Indeed, it has been described that the Rosaceae family origin comes from a polyploidization event, explaining the presence of two of these genes in the Rosaceae species [37]. In agreement, *A. thaliana* presents three copies of *FLS* and four of *PAL*, as result of the two polyploidization events that originated this species [38,39]. Functional redundancy and natural selection lead to gene loss, silencing or neo-functionalization [40]. Dosage-dependent genes are usually retained in the duplicated genomes [41], suggesting the dosage dependence of *FLS* and *PAL* in the phenylpropanoid pathway.

Taking into account this information, we did a screening of possible MRE *cis-acting* elements involved in phenolic biosynthesis. Results revealed a common MRE (MYBCORE) containing also a BOXLCOREDCPAL motif in *ParDFR* and *ParPAL2,* which suggested that both genes can be regulated by the same transcription factor. However, this MRE was not found in *ParPAL1*. This fact suggests different regulation or even different roles of each identified *PAL* paralogues in apricot. This is also supported by the high correlation of *ParDFR* and *ParPAL2* expression (Table 4), which indicates that they share the same regulation and supports the existence of different regulation for each paralogue. This specialization between paralogues that result from ancestral genomic duplications has been previously described [42] and even can lead to neo-functionalization of genes. In addition, most of the accessions with a high expression for *ParFLS2,* such as ‘Dama Rosa’, are siblings of the traditional cultivar ‘Ginesta’, a cultivar that had more than 50% of red-blush [34]. These results suggest a possible role of *ParFLS2* in anthocyanin synthesis, in agreement with previous studies that proposed a disequilibrium in the expression of *FLS* and *DFR* enzymes determine the accumulation of flavonols and anthocyanins [20,21,30].

The transcriptional study was made at fruit maturity. From the results obtained, a further analysis of *ParPAL1* in different immature fruit stages would contribute to identify accurately its role in peel polyphenol content. Furthermore, the results obtained indicated a possible shared regulation for *ParFLS2* and *ParDFR* expression related to anthocyanin biosynthesis in apricot. Our results contribute to unravel the relationship between genetic of red-blush trait and polyphenol compounds and the relationship between *ParFLS2* and anthocyanin biosynthesis in apricot.

## 4. Materials and Methods

### 4.1. Plant Material

A set of 2 Mediterranean cultivars (‘Canino’ and ‘Mitger’) a North American variety (‘Goldrich’) and 9 hybrids from the IVIA’s apricot breeding program were analyzed (Table 5). ‘Goldrich’ used as the main donor of resistance to PPV at the breeding program is one of the parents in most of the resistant hybrids obtained. ‘Canino’ and ‘Mitger’ are two autochthonous varieties used for introgression of adaptability to Mediterranean conditions. The trees are maintained at the IVIA’s apricot collection located in Moncada (latitude 37°45′31.5″ N, longitude 1°01′35.1″ W), Spain.

Five fruits per tree were harvested at the ripening stage during two growing seasons (2019 and 2020). For each fruit, the peel was separated from the flesh with a peeler. The samples consisted of a mix of the peel from 5 fruits per genotype and year. Samples were frozen with liquid nitrogen and kept at −80 °C until processing.

### 4.2. HPLC Analysis

For HPLC analysis, the tissue was processed to lyophilized powder. Tissue homogenization was carried out using a vortex. Phenolic compounds were extracted and determined according to the procedure described by [43,44]. Briefly, 10 mg of freeze-dried peel were mixed with 1 mL of DMSO/MeOH (1:1, *v*/*v*). Then, the sample was centrifuged (Eppendorf 5810R centrifuge; Eppendorf Iberica, Madrid, Spain) at 4 °C for 20 min at 10,000 rpm. The supernatant was filtered through a 0.45 μm nylon filter and analyzed by HPLC-DAD and HPLC-MS in a reverse-phase column C18 Tracer Excel 5 μm 120 OSDB (250 mm × 4.6 mm) (Teknokroma, Barcelona, Spain). An Alliance liquid chromatographic system (Waters, Barcelona, Spain) equipped with a 2695 separation module, was coupled to a 2996 photodiode array detector and a ZQ2000 mass detector. A gradient mobile phase consisting of acetonitrile (solvent A) and 0.6% acetic acid (solvent B) was used at a flow rate of 1 mL/min, with an injection volume of 10 μL. The gradient change was as follows: 10% 2 min, 10–75% 28 min, 75–10% 1 min, and hold at 10% 5 min. An HPLC-MS analysis was performed and worked under electrospray ion positive (flavonoids) and negative (phenolic acids) conditions. Capillary voltage was 3.50 kV, cone voltage was 20 V, source temperature was 100 °C, desolvation temperature was 225 °C, cone gas flow was 70 L/h.

Chromatograms were recorded at 340 nm absorbance. Chlorogenic acid and rutin were identified by comparison with pure standards obtained from Sigma-Aldrich (Sigma Co., Barcelona, Spain) using an external calibration curve. In addition, standards were run daily with samples for validation. Neochlorogenic acid and quercetin-3-glucuronide were tentatively identified based on their retention times, UV-vis spectra and mass spectrum characteristics and mass spectrum data with available data described in the literature. For the quantitative analysis, an external calibration curve with available standards chlorogenic acid and rutin was carried out. In addition, standards were run daily with samples for validation. All the solvents used were of LC-MS grade. Three samples per cultivar were analyzed and all the samples were run in triplicate. The Empower 2 software (Waters, Spain) was used for data processing. Standard measurements (Figure S1) and a sample of the chromatograms in apricot peel sample (Figure S2) are included.

### 4.3. Obtention of Gene Sequences and Cis-Acting Elements Motif Identification

To identify the genetic regulation in the phenolics biosynthesis pathway, a set of genes encoding for dihydroflavonol-4-reductase (DFR), flavonol synthase (FLS) and phenylalanine ammonia-lyase (PAL) were selected. To obtain putative orthologs of apricot species, a BLAST search was performed using *A. thaliana* and *P. persica* described genes in GDR (Genome Database of Rosaceae) [45] on *Prunus armeniaca* genome.

Identification of cis-acting elements was made from a total sequence of 1500 bp upstream of the start codons from the *Prunus armeniaca* genome published at Genomic Database of Rosaceae (GDR). Analysis of cis-acting elements was made using PLACE (Plant cis-acting Elements) database [46] and searching for described motifs related to the phenolic pathway.

In addition, to check the sequence conservation among species, a phylogenetic analysis was made with the obtained *Prunus armeniaca* genes predicted proteins and *Prunus persica* (*PpeDFR* (Prupe.1G376400.1), *PpeFLS1* (Prupe.1G502700.1), *PpeFLS2* (Prupe.1G502800.1), *PpePAL1* (ppa002328m)*, PpePAL2* (ppa002099m)), *Fragaria vesca* (*FvDFR* (mrna15174.1-v1.0-hybrid)*, FvFLS1* (mrna11126.1-v1.0-hybrid), *FvPAL1* (mrna23261.1-v1.0-hybrid), *FvPAL2* (mrna09753.1-v1.0-hybrid)), *Vitis vinifera* (*VvDFR* (GSVIVT01009742001)*, VvFLS1*(*GSVIVT01008913001*), *VvPAL1* (GSVIVT01016257001)), *Malus domestica* (*MdDFR* (MDP0000734274), *MdFLS1* (MDP0000311541), *MdFLS2* (MDP0000294667), *MdPAL1* (MDP0000668828), *MdPAL2* (MDP0000261492)) and *Arabidopsis thaliana* (*AtDFR* (NM_123645.4), *AtFLS1* (U84259.1), *AtFLS2* (BT003134.1)*, AtFLS3* (NM_125754.3), *AtPAL1* (AY303128.1), *AtPAL2* (AY303129.1), *AtPAL3* (NM_001203294.1), *AtPAL4* (AY303130.1)) predicted proteins. For apricot, coding sequences (*ParDFR* (PARG07267m); *ParPAL1* (PARG18722m), *ParPAL2* (PARG02214m), *ParFLS1* (PARG08425m), *ParFLS2* (PARG08426m), were translated into proteins with a DNA translate tool from *Expasy* [47]. Multiple protein sequence alignment was performed with the *ClustalW* program with *MEGA X* v.10.1.8 software [48], and a phylogenetic tree was built with the Neighbor-Joining method using *MEGA X* v.10.1.8 software with a bootstrap value of 1000 replicates.

The number of amino acid differences per site from between sequences (*p-distance*) was calculated with MEGA X Software with bootstrap method with 1000 replications. 1- *p-distance* was calculated to similarity estimation among proteins. In addition, a BLAST and a synteny of *Prunus persica* against and *Prunus armeniaca* reference genome was performed in the GDR database. Moreover, a BLAST of *Arabidopsis thaliana* against *Prunus armeniaca* genome was also performed in GDR database [45].

### 4.4. Gene Expression

Samples consisted of 80 mg of powered tissue. RNA isolation was made using *Plant/Fungi Total RNA Purification Kit* (NORGEN, Thorold, ON, Canada) with some modifications. Frozen power tissue was diluted in 600 mL of lysis buffer C, a 2% PVP-40 and 2% β-mercaptoethanol was added. Purified RNA quality and integrity were checked by agarose gel electrophoresis, RNA was quantified by Qubit (Invitrogen, Carlsbad, CA, USA).

cDNA synthesis was obtained from 500 ng of RNA diluted in 10 μL reaction using the *PrimeScript RT Reagent* kit (‘Perfect Real Time’) (Takara Bio, Otsu, Japan).

Amplification was carried out with StepOnePLus Real-Time PCR System (Life Technologies, Carlsbad, CA, USA) software and TB Green Premix Ex Taq (Tli RNaseH Plus) (Takara Bio, Otsu, Japan) kit was used. Mix reaction contained 7.5 μL enzyme, 0.09 μL of primers (100 μM), 0.3 mL ROX, 5.02 μL H20, and 1 μL of cDNA. Mix was incubated at 95 °C for 30 s, followed by 40 cycles of 5 s at 95 °C and 30 s at 60 °C. Finally, the mix was incubated for 15 s at 95 °C, followed by a minute at 60 °C and 15 s at 95 °C. Apricot *ACTIN* and *SAND* geometric mean expression was used as housekeeping gene for normalization. Primers used are indicated in Table 6. For each year and genotype, the calculated expression was the mean of three biological replicates. Relative expression of each gene was calculated using the relative standard curve method.

### 4.5. Data Analysis

Data were statistically analyzed by *Statgraphics Centurion VII* version 17.2.00 software (Statpoint Technologies Inc., Warrenton, VA, USA). Differences among samples and years were analyzed with the Kruskal-Wallis test (*p* ≤ 0.05) and averages were compared using the Multiple Range Test with Bonferroni method.

For testing the contribution of ‘Goldrich’ to the phenolic content and genetic expression in the set of accessions, we performed a regression of the data to a general linear model [8]. In the model, the phenotype is linearly explained as follows:

Phenotype = C + G_Goldrich_ + Year + G_Goldrich_ × Year + Residual.

where C is the general average of the population (constant), G_Goldrich_ is the genetic effect of ‘Goldrich’, Year is the environmental effect due to the year and Residual is the residual effect. The model was calculated using the *Statgraphics Centurion VII* version 17.2.00 software (Statpoint Technologies, Warrenton, VA, USA). A quantitative variable for evaluating the genetic effect of ‘Goldrich’ was included with a value of 1 for ‘Goldrich’, 0.5 value for ‘Goldrich × X’ hybrids, and a null value for the other genotypes non-related to ‘Goldrich’. Model parameters were estimated with a 95% confidence level (*p* ≤ 0.05).

Elucidation of parameters significantly influent in phenolic content was made by a linear regression model with *Statgraphics Centurion VII* version 17.2.00 software (Statpoint Technologies, Warrenton, VA, USA). Parameters included in the linear regression were: genetic expression in apricot of *ParDFR*, *ParFLS1*, *ParFLS2*, *ParPAL1*, and *ParPAL2*, and the following genetic expression ratios: *ParPAL1/ParPAL2*, *ParPAL1*/*ParFLS1*, *ParPAL1/ParFLS2*, *ParPAL2/ParFLS1*, *ParPAL2/ParFLS2,* and *ParFLS1/ParFLS2*. Non-significant parameters were excluded from each model and only those significant were maintained.

In addition, a multivariate analysis was performed with *Statgraphics XVII* software (Statpoint Technologies, Warrenton, VA, USA) to study Pearson correlation among gene expression, phenolic contents, and the relationships among all of them. Correlation with a *p* < 0.05 was considered significant.

Graphics were made using R-studio software (Version 1.1.463, 2009–2018, Rstudio, Inc., Boston, MA, USA) with ‘stats’, grDevices’, and ‘graphics’ (R Core Team), ‘dplyr’ [49], ‘readxl’ [50], ‘plyr’ [51], ‘scales’ [52] and ‘ggplot2’ [53] packages.

## 5. Conclusions

The set of accessions studied showed the levels of expression of key genes in the polyphenol biosynthesis pathway are genotype-dependent. In addition, cultivar ‘Goldrich’, used as donor of PPV resistance, contributed positively to *ParPAL1* expression levels. This genetic expression agrees with the previously described contribution to total polyphenol content. Transcriptional data of the main genes involved in critical points at the polyphenol pathway have been described and their relationships with the different polyphenol compounds identified. Higher expression of *ParDFR* and *ParPAL2* has been associated to red-blushed accessions. Differences in expression between paralogues in the phenolic pathway can be linked to the presence of a *BOXCOREDLPAL* cis-acting element related to the genes involved in anthocyanin synthesis: *ParDFR*, *ParFLS2*, and *ParPAL2.*

## Figures and Tables

**Table 1:** Polyphenol total content (mg/100 g DW). Average ± standard deviation. Different letter means significant differences among genotypes. Varieties with * produced fruits with a red-blush on the skin >50%.

Genotype	2019	2020	Two-Years Average
Canino	539.63 ± 12.81 ab	669.52 ± 30.03 a	604.58 ± 74.08 a
Dama Rosa *	1725.21 ± 222.12 g	1565.50 ± 64.26 e	1645.36 ± 170.41 d
Dama Taronja *	699.71 ± 27.00 bcd	1171.19 ± 286.97 cd	935.45 ± 316.10 abc
GG9310 *	1024.98 ± 9.58 ef	1008.08 ± 80.72 abc	1016.53 ± 52.23 bc
GG979 *	630.10 ± 11.57 abc	1018.73 ± 159.22 bc	824.41 ± 235.59 abc
Goldrich	894.02 ± 25.15 de	876.18 ± 22.06 abc	885.10 ± 23.31 abc
GP9817 *	1167.16 ± 17.91 f	916.08 ± 67.15 abc	1041.62 ± 144.38 bc
HG9821	514.38 ± 3.73 ab	814.96 ± 51.46 ab	664.67 ± 167.84 ab
HG9850	422.75 ± 18.96 a	695.63 ± 39.43 ab	559.19 ± 152.00 a
HM964 *	822.83 ± 28.91 cde	825.43 ± 23.56 ab	824.13 ± 23.63 abc
Mitger	832.44 ± 28.91 cde	1509.14 ± 76.83 de	1170.79 ± 374.27 c
SEOP934	497.04 ± 20.18 ab	738.04 ± 75.04 ab	617.54 ± 140.85 a

**Table 2:** ‘Goldrich’ contribution to phenolic content: Sum of squares (SS) and model parameters coefficients. *SS_r_*: SS relative; *SS_t_*: SS total; *p-v*: *p*-value; *G_r_*: Goldrich relative; *Sig*: Significance.

	Year			Goldrich			Year × Goldrich			Residual			
	*SS_t_*	*SS_r_*	*p-v*	*SS_t_*	*SS_r_*	*p-v*	*SS_t_*	*SS_r_*	*p-v*	*SS_t_*	*SS_r_*	Total	R2
**Neochlogenic**	108,653	0.095	0.0039	120,718	0.1051	0.0024	10.0892	0.000	0.9771	826,863	0.720	1.15 × 10^6^	0.280
**Chlorogenic**	288,022	0.149	0.0004	134,852	0.0700	0.0124	12,565.3	0.007	0.4359	1.39 × 10^6^	0.722	1.93 × 10^6^	0.278
**Rutin**	1140.39	0.001	0.8023	90,359.6	0.0596	0.0286	92,447.4	0.061	0.0269	1.23 × 10^6^	0.811	1.51 × 10^6^	0.189
**Quercetin-3-glucurunide**	26.687	0.001	0.7803	523.664	0.0208	0.219	605.821	0.024	0.1865	23,134.6	0.921	25,129.6	0.079
**Total content**	684,536	0.083	0.0089	1.08 × 10^6^	0.1312	0.0012	191,481	0.023	0.1591	6.42 × 10^6^	0.782	8.21 × 10^6^	0.218
			**Constant**			**Goldrich**							
			**Mean**	**Lower Lim**	**Error**	**Mean**	**Lower Lim**	**Error**	***G_r_***	***Sig***			
**Neochlogenic**			228.557	193.471	35.086	127.94	46.913	81.027	56.0%	**			
**Chlorogenic**			236.938	191.437	45.501	135.222	30.1403	105.082	57.1%	*			
**Rutin**			296.716	253.959	42.757	110.689	11.9464	98.743	37.3%	*			
**Quercetin-3-glucurunide**			25.4043	19.5356	5.869	8.4265	−5.12673	13.553	33.2%	NS			
**Total content**			787.615	689.815	97.800	382.278	156.418	225.860	48.5%	**			

* Significant differences (*p* ≤ 0.05); ** Significant differences (*p* ≤ 0.01); NS: non-significant.

**Table 3:** ‘Goldrich’ contribution to genetic expression: Sum of squares and model parameters coefficients. *SS_r_*: SS relative; *SS_t_*: SS total; *p-v*: *p*-value; *G_r_*: Goldrich relative; *Sig*: Significance.

	Year			Goldrich			Year × Goldrich			Residual		Total	R2
	*SS_t_*	*SS_r_*	*p-v*	*SS_t_*	*SS_r_*	*p-v*	*SS_t_*	*SS_r_*	*p-v*	*SS_t_*	*SS_r_*		
*ParDFR*	2.2294	0.023	0.1686	17.738	0.1795	0.0002	2.4002	0.024	0.1534	78.2977	0.792	98.8062	0.208
*ParFLS1*	17.9388	0.073	0.002	8.0677	0.0327	0.0362	11.5896	0.047	0.0124	219.4	0.888	246.939	0.112
*ParFLS2*	0.0474	0.000	0.8526	6.7223	0.0636	0.0297	2.8529	0.027	0.1527	92.7271	0.878	105.665	0.122
*ParPAL1*	0.3709	0.006	0.5249	4.2923	0.0655	0.0332	0.3443	0.005	0.5401	60.8265	0.928	65.5523	0.072
*ParPAL2*	1.0437	0.009	0.4138	16.03	0.1310	0.0020	0.1313	0.001	0.7714	104.974	0.859	122.217	0.141
			**Constant**			**Goldrich**							
			**Mean**	**Lower Lim**	**Error**	**Mean**	**Lower Lim**	**Error**	***G_r_***	***Sig***			
*ParDFR*			1.57183	1.2304	0.341	−1.5509	−2.3393	0.788	−98.7%	**			
*ParFLS1*			1.54347	1.2259	0.318	−0.7697	−1.4891	0.719	−49.9%	*			
*Par* *FLS2*			1.64119	1.2696	0.372	−0.9547	−1.8128	0.858	−58.2%	*			
*ParPar* *PAL1*			1.08334	0.7799	0.303	0.7652	0.0628	0.702	70.6%	*			
*ParPAL2*			1.80279	1.4075	0.395	−1.4731	−2.3861	0.913	−81.7%	**			

* Significant differences (*p* ≤ 0.05); ** Significant differences (*p* ≤ 0.01); NS: non-significant.

**Table 4:** Pearson correlation coefficients among compounds and gene expression.

Parameter	*ParPAL1*	*ParPAL2*	*ParDFR*	*ParFLS1*	*ParFLS2*	Neochlorogenic	Chlorogenic	Rutin
*ParPAL1*								
*ParPAL2*	0.1507							
*ParDFR*	−0.0163	0.8098 **						
*ParFLS1*	−0.1899	0.3408 **	0.3139 **					
*ParFLS2*	−0.2726 *	0.0273	0.1629	0.1261				
Neochlorogenic	−0.1951	−0.1369	−0.0258	−0.1043	0.2919 *			
Chlorogenic	−0.1317	−0.1216	−0.017	0.0051	−0.011	0.6835 **		
Rutin	0.0283	0.1635	0.0568	−0.2062	0.1943	0.2929 *	0.0734	
Quercetin-3-glucuronide	−0.1638	0.0083	0.0477	−0.144	0.1216	0.4452 **	0.2233	0.7407 **

* Significant differences (*p* ≤ 0.05); ** Significant differences (*p* ≤ 0.01).

**Table 5:** Plant material used in the study, pedigree, and origin.

Genotype	Pedigree	Origin
Canino	Unknown	Spain
Dama Rosa	Goldrich × Ginesta	IVIA
Dama Taronja	Goldrich × Katy	IVIA
GG9310	Goldrich × Ginesta	IVIA
GG979	Goldrich × Ginesta	IVIA
Goldrich	Sunglo × Perfection	USA
GP9817	Goldrich × Palau	IVIA
HG9821	Harcot × Ginesta	IVIA
HG9850	Harcot × Ginesta	IVIA
HM964	Harcot × Mitger	IVIA
Mitger	Unknown	Spain
SEOP934	SEO × Palau	IVIA

**Table 6:** Genes and primers.

Gen	Forward	Reverse
*ParPAL1*	CGACTGGGTTATGGATAGCATGA	CAATGTGTGGGTAGATTCTGTGC
*ParPAL2*	TAAAGAGGTGGATAGTGCAAGGG	GAGAACACCTTGTCGCATTCTTC
*ParFLS1F*	TGGAGGGGATGACATGGTTTATC	CCGTTGCTCATAATCTCCATCTG
*ParFLS2F*	ACAGGAGGAAAAGGAGGCTTATG	GGCCAGAACCGGTAATTAATGAC
*ParDFR*	GTTCGAAGGCTGGTGTTTACATC	GAGAAATGGGCCAATCACAAGAG
*ACTIN*	CTTCTTACTGAGGCACCCCTGAAT	AGCATAGAGGGAGAGAACTGCTTG
*SAND*	TCGTGGGTACCAGGAAAACGACAT	CCTGCTAGCTTGTGTTCATCTCCA

## Data Availability

All data are available in the MS and supplementary data.

## Supplementary Materials

The following are available online at https://www.mdpi.com/article/10.3390/ijms22073411/s1, Table S1. Polyphenol content (mg/100 g DW) in chlorogenic acid (A), neochlorogenic acid (B), rutin (C) and quercetin-3-glucuronide (D). Table S2. *Prunus persica* and *Prunus armeniaca* synteny and protein identity. Table S3: *Arabidopsis thaliana* and *Prunus armeniaca* protein identity. Table S4: p-distance for PAL (A), DFR (B) and FLS (C) proteins. Table S5: Similarity (1-p-distance) among protein sequences of PAL (A), DFR (B), FLS (C). Table S6: Genetic expression of studied genotypes. Different letter means significant differences among genotypes. Table S7: Linear regression model in caffeate-derivates content. Table S8: Linear regression model in flavonols content. Figure S1: Standard Chromatogram and retention times. Figure S2: ‘Canino’ chromatogram.

## References

[1] Bailey C.H., Hough L.F. (1975). Advances in Fruit Breeding.

[2] Roussos P.A., Sefferou V., Denaxa N.-K., Tsantili E., Stathis V. (2011). Apricot (*Prunus armeniaca* L.) fruit quality attributes and phytochemicals under different crop load. Sci. Hortic..

[3] Ruiz D., Egea J. (2007). Phenotypic diversity and relationships of fruit quality traits in apricot (*Prunus armeniaca* L.) germplasm. Euphytica.

[4] Ferrer J.-L., Austin M., Stewart C., Noel J. (2008). Structure and function of enzymes involved in the biosynthesis of phenylpropanoids. Plant Physiol. Biochem..

[5] Harborne J.B. (2009). Plant Secondary Metabolism. Plant Ecology.

[6] Bizjak J., Mikulic-Petkovsek M., Stampar F., Veberic R. (2013). Changes in Primary Metabolites and Polyphenols in the Peel of “Braeburn” Apples (*Malus domestica* Borkh.) during Advanced Maturation. J. Agric. Food Chem..

[7] Campbell O.E., Padilla-Zakour O.I. (2013). Phenolic and carotenoid composition of canned peaches (Prunus persica) and apricots (*Prunus armeniaca*) as affected by variety and peeling. Food Res. Int..

[8] Gómez-Martínez H., Bermejo A., Zuriaga E., Badenes M. (2021). Polyphenol content in apricot fruits. Sci. Hortic..

[9] Fu L., Xu B.-T., Xu X.-R., Qin X.-S., Gan R.-Y., Li H.-B. (2010). Antioxidant Capacities and Total Phenolic Contents of 56 Wild Fruits from South China. Molecules.

[10] Gan R.-Y., Deng Z.-Q., Yan A.-X., Shah N.P., Lui W.-Y., Chan C.-L., Corke H. (2016). Pigmented edible bean coats as natural sources of polyphenols with antioxidant and antibacterial effects. LWT.

[11] Mokrani A., Krisa S., Cluzet S., Da Costa G., Temsamani H., Renouf E., Mérillon J.-M., Madani K., Mesnil M., Monvoisin A. (2016). Phenolic contents and bioactive potential of peach fruit extracts. Food Chem..

[12] Erdogan-Orhan I., Kartal M. (2011). Insights into research on phytochemistry and biological activities of *Prunus armeniaca* L. (apricot). Food Res. Int..

[13] Fraser C.M., Chapple C. (2011). The Phenylpropanoid Pathway in *Arabidopsis*. Arab. Book.

[14] Raes J., Rohde A., Christensen J.H., Van De Peer Y., Boerjan W. (2003). Genome-Wide Characterization of the Lignification Toolbox in Arabidopsis. Plant Physiol..

[15] Fukasawa-Akada T., Kung S.-D., Watson J.C. (1996). Phenylalanine ammonia-lyase gene structure, expression, and evolution in *Nicotiana*. Plant Mol. Biol..

[16] Reichert A.I., He X.-Z., Dixon R.A. (2009). Phenylalanine ammonia-lyase (PAL) from tobacco (*Nicotiana tabacum*): Characterization of the four tobacco *PAL* genes and active heterotetrameric enzymes. Biochem. J..

[17] Hamberger B., Ellis M., Friedmann M., Souza C.D.A., Barbazuk B., Douglas C.J. (2007). Genome-wide analyses of phenylpropanoid-related genes in *Populus trichocarpa*, *Arabidopsis thaliana*, and *Oryza sativa*: The Populus lignin toolbox and conservation and diversification of angiosperm gene familiesThis article is one of a selection of papers published in the Special Issue on Poplar Research in Canada. Can. J. Bot..

[18] Irisarri P., Zhebentyayeva T., Errea P., Pina A. (2016). Differential expression of phenylalanine ammonia lyase (PAL) genes implies distinct roles in development of graft incompatibility symptoms in *Prunus*. Sci. Hortic..

[19] Pelletier M.K., Burbulis I.E., Winkel-Shirley B. (1999). Disruption of specific flavonoid genes enhances the accumulation of flavonoid enzymes and end-products in *Arabidopsis* seedlings. Plant Mol. Biol..

[20] Owens D.K., Alerding A.B., Crosby K.C., Bandara A.B., Westwood J.H., Winkel B.S. (2008). Functional Analysis of a Predicted Flavonol Synthase Gene Family in Arabidopsis. Plant Physiol..

[21] Kuhn B.M., Geisler M., Bigler L., Ringli C. (2011). Flavonols Accumulate Asymmetrically and Affect Auxin Transport in *Arabidopsis*. Plant Physiol..

[22] Martens S., Knott J., Seitz C.A., Janvari L., Yu S.-N., Forkmann G. (2003). Impact of biochemical pre-studies on specific metabolic engineering strategies of flavonoid biosynthesis in plant tissues. Biochem. Eng. J..

[23] Shimada N., Sasaki R., Sato S., Kaneko T., Tabata S., Aoki T., Ayabe S.-I. (2005). A comprehensive analysis of six dihydroflavonol 4-reductases encoded by a gene cluster of the *Lotus japonicus* genome. J. Exp. Bot..

[24] Lopiero A., Puglisi I., Petrone G. (2006). Gene characterization, analysis of expression and in vitro synthesis of dihydroflavonol 4-reductase from [*Citrus sinensis* (L.) Osbeck]. Phytochemistry.

[25] Xie D.-Y., Jackson L.A., Cooper J.D., Ferreira D., Paiva N.L. (2004). Molecular and Biochemical Analysis of Two cDNA Clones Encoding Dihydroflavonol-4-Reductase from *Medicago truncatula*. Plant Physiol..

[26] Singh K., Kumar S., Yadav S.K., Ahuja P.S. (2008). Characterization of dihydroflavonol 4-reductase cDNA in tea [*Camellia sinensis* (L.) O. Kuntze]. Plant Biotechnol. Rep..

[27] Huang Y., Gou J., Jia Z., Yang L., Sun Y., Xiao X., Song F., Luo K. (2012). Molecular Cloning and Characterization of Two Genes Encoding Dihydroflavonol-4-Reductase from *Populus trichocarpa*. PLoS ONE.

[28] Hartmann U., Sagasser M., Mehrtens F., Stracke R., Weisshaar B. (2005). Differential combinatorial interactions of cis-acting elements recognized by R2R3-MYB, BZIP, and BHLH factors control light-responsive and tissue-specific activation of phenylpropanoid biosynthesis genes. Plant Mol. Biol..

[29] Jin X., Huang H., Wang L., Sun Y., Dai S. (2016). Transcriptomics and Metabolite Analysis Reveals the Molecular Mechanism of Anthocyanin Biosynthesis Branch Pathway in Different *Senecio cruentus* Cultivars. Front. Plant Sci..

[30] Eluo P., Ening G., Ewang Z., Eshen Y., Ejin H., Eli P., Ehuang S., Ezhao J., Ebao M. (2016). Disequilibrium of Flavonol Synthase and Dihydroflavonol-4-Reductase Expression Associated Tightly to White vs. Red Color Flower Formation in Plants. Front. Plant Sci..

[31] Dossett M., Lee J., Finn C.E. (2011). Characterization of a novel anthocyanin profile in wild black raspberry mutants: An opportunity for studying the genetic control of pigment and color. J. Funct. Foods.

[32] Kayesh E., Shangguan L., Korir N.K., Sun X., Bilkish N., Zhang Y., Han J., Song C., Cheng Z.-M., Fang J. (2013). Fruit skin color and the role of anthocyanin. Acta Physiol. Plant..

[33] Wang K., Jin P., Han L., Shang H., Tang S., Rui H., Duan Y., Kong F., Kai X., Zheng Y. (2014). Methyl jasmonate induces resistance against *Penicillium citrinum* in Chinese bayberry by priming of defense responses. Postharvest Biol. Technol..

[34] Badenes M.L., Martínez-Calvo J., Gómez H., Zuriaga E. (2018). ‘Dama Taronja’ and ‘Dama Rosa’ Apricot Cultivars that are Resistant to Sharka (Plum pox virus). HortScience.

[35] Bureau S., Renard C.M., Reich M., Ginies C., Audergon J.-M. (2009). Change in anthocyanin concentrations in red apricot fruits during ripening. LWT.

[36] Roulin A., Auer P.L., Libault M., Schlueter J., Farmer A., May G., Stacey G., Doerge R.W., Jackson S.A. (2012). The fate of duplicated genes in a polyploid plant genome. Plant J..

[37] Xiang Y., Huang C.-H., Hu Y., Wen J., Li S., Yi T., Chen H., Xiang J., Ma H. (2016). Evolution of Rosaceae Fruit Types Based on Nuclear Phylogeny in the Context of Geological Times and Genome Duplication. Mol. Biol. Evol..

[38] Bomblies K., Weigel D. (2007). *Arabidopsis*—A model genus for speciation. Curr. Opin. Genet. Dev..

[39] Bomblies K., Madlung A. (2014). Polyploidy in the *Arabidopsis* genus. Chromosom. Res..

[40] Del Pozo J.C., Ramirez-Parra E. (2015). Whole genome duplications in plants: An overview from *Arabidopsis*. J. Exp. Bot..

[41] Edger P.P., Pires J.C. (2009). Gene and genome duplications: The impact of dosage-sensitivity on the fate of nuclear genes. Chromosom. Res..

[42] Lian S., Zhou Y., Liu Z., Gong A., Cheng L. (2020). The differential expression patterns of paralogs in response to stresses indicate expression and sequence divergences. BMC Plant Biol..

[43] Cano A., Medina A., Bermejo A. (2008). Bioactive compounds in different citrus varieties. Discrimination among cultivars. J. Food Compos. Anal..

[44] Cano A., Bermejo A. (2011). Influence of rootstock and cultivar on bioactive compounds in citrus peels. J. Sci. Food Agric..

[45] Genome Dabase of Rosaceae. https://www.rosaceae.org/.

[46] Higo K., Ugawa Y., Iwamoto M., Korenaga T. (1999). Plant cis-acting regulatory DNA elements (PLACE) database: 1999. Nucleic Acids Res..

[47] Expasy. https://web.expasy.org/translate/.

[48] Kumar S., Stecher G., Li M., Knyaz C., Tamura K. (2018). MEGA X: Molecular Evolutionary Genetics Analysis across computing platforms. Mol. Biol. Evol..

[49] Wickham H. (2020). Package ‘Dplyr’. https://dplyr.tidyverse.org.

[50] Wickham H. Readxl: Read Excel Files. 2016; pp. 1–9. https://readxl.tidyverse.org.

[51] Wickham H. (2020). Package ‘Plyr’. http://had.co.nz/plyr.

[52] Wickham H., Seidel D. (2020). Package ‘Scales’. https://scales.r-lib.org.

[53] Wickham H. (2009). Ggplot2.

