# Supplementary material for: Insights of Phenolic Pathway in Fruits: Transcriptional and Metabolic Profiling in Apricot (Prunus armeniaca)
Source: Int J Mol Sci. 2021 Mar 26;22(7):3411. doi: 10.3390/ijms22073411 (PMC8037730; doi:10.3390/ijms22073411)
Supplement: Supplementary file 1 [file ijms-22-03411-s001.pdf]

Supplementary materials

Table S1. Polyphenol content (mg/100gDW) in chlorogenic acid (A), neochlorogenic acid (B), rutin (C) and quercetin-3-glucuronide (D).

A

Genotype	Chlorogenic acid								
	2019			2020			Two years average		
	Mean	Sd	Significance	Mean	Sd	Significance	Mean	Sd	Significance
Canino	103,82	3,77	abc	103,02	3,83	a	103,42	3,43	a
Dama Rosa	525,47	54,31	f	727,48	22,50	f	626,48	116,73	d
Dama Taronja	95,05	5,44	ab	225,55	52,91	b	160,30	79,00	abc
GG9310	93,80	2,26	ab	276,97	20,84	bc	185,39	101,20	abc
GG979	276,77	6,35	de	432,58	70,34	de	354,67	96,33	c
Goldrich	270,33	5,87	d	339,74	9,26	cd	305,03	38,65	abc
GP9817	322,07	5,47	e	338,27	25,23	cd	330,17	18,58	bc
HG9821	148,30	1,14	c	405,31	23,07	d	276,81	141,53	abc
HG9850	77,85	3,13	a	199,56	10,33	ab	138,71	67,02	ab
HM964	236,15	5,93	d	422,72	10,24	d	329,43	102,46	bc
Mitger	147,31	4,06	c	524,40	26,66	e	335,86	207,24	bc
SEOP934	137,24	4,99	bc	203,33	21,56	b	170,29	38,81	abc

B

Genotype	Neochlorogenic acid								
	2019			2020			Two years average		
	Mean	Sd	Significance	Mean	Sd	Significance	Mean	Sd	Significance
Canino	190,79	3,40	cd	167,15	4,92	ab	178,97	13,49	ab
Dama Rosa	418,98	40,09	f	533,02	18,74	d	476,00	68,45	d
Dama Taronja	205,53	7,25	cd	424,93	95,70	d	315,23	134,64	bcd
GG9310	380,90	2,50	f	459,17	33,65	d	420,03	47,88	cd
GG979	183,82	18,58	c	288,73	41,44	c	236,27	64,24	ab
Goldrich	190,45	4,22	cd	276,43	3,86	c	233,44	47,23	ab
GP9817	232,33	21,43	d	290,09	18,47	c	261,21	36,34	abc
HG9821	168,36	0,48	c	274,32	16,91	bc	221,34	59,02	ab
HG9850	71,19	2,30	a	156,55	5,29	a	113,87	46,90	a
HM964	286,94	7,09	e	214,70	4,89	abc	250,82	39,94	abc
Mitger	119,98	4,42	b	504,88	21,29	d	312,43	211,27	bcd
SEOP934	106,42	3,70	ab	235,29	27,66	abc	170,85	72,76	ab

C

Genotype	2019			2020			Two years average		
	**Mean**	**Sd**	**Significance**	**Mean**	**Sd**	**Significance**	**Mean**	**Sd**	**Significance**
Canino	231,84	6,15	a	380,05	20,16	cde	305,95	82,26	abcd
Dama Rosa	696,75	115,40	f	262,81	19,49	bc	479,78	248,94	cd
Dama Taronja	344,06	14,34	bc	457,26	120,96	e	400,66	98,89	bcd
GG9310	510,34	2,52	de	259,25	24,30	bc	384,80	138,40	abcd
GG979	158,85	3,34	a	283,25	44,86	bc	221,05	73,84	ab
Goldrich	415,42	14,03	cd	249,89	14,45	abc	332,65	91,55	abcd
GP9817	575,79	1,57	e	271,24	22,95	bc	423,51	167,45	bcd
HG9821	186,99	1,90	a	125,81	10,89	a	156,40	34,23	a
HG9850	250,31	11,94	ab	317,01	23,57	cd	283,66	40,17	abcd
HM964	268,38	14,34	ab	163,78	8,28	ab	216,08	58,24	ab
Mitger	533,10	25,53	e	445,09	25,85	de	489,10	53,40	d
SEOP934	232,64	11,91	a	276,09	27,71	bc	254,36	30,50	abc

Table title row: **Rutin**

D

Quercetin-3-glucuronide									
**Genotype**	**2019**			**2020**			**Two years average**		
	**Mean**	**Sd**	**Significance**	**Mean**	**Sd**	**Significance**	**Mean**	**Sd**	**Significance**
Canino	13,18	0,64	a	19,30	1,18	ab	16,24	3,46	ab
Dama Rosa	84,01	12,43	g	42,18	3,78	c	63,09	24,34	c
Dama Taronja	55,08	1,33	f	63,44	17,45	d	59,26	11,98	c
GG9310	39,93	7,55	e	12,70	1,95	a	26,31	15,71	ab
GG979	10,66	0,38	a	14,17	2,70	a	12,41	2,58	a
Goldrich	17,82	1,33	ab	10,11	0,28	a	13,97	4,31	ab
GP9817	36,97	0,39	de	16,49	2,23	a	26,73	11,31	ab
HG9821	10,73	0,33	a	9,51	0,75	a	10,12	0,85	a
HG9850	23,42	1,65	abcd	22,51	1,86	ab	22,96	1,65	ab
HM964	31,36	1,66	bcde	24,23	1,40	ab	27,80	4,14	ab
Mitger	32,04	1,68	cde	34,77	3,34	bc	33,41	2,80	b
SEOP934	20,74	1,15	abc	23,33	3,89	ab	22,04	2,93	ab

Table S2. *Prunus persica* and *Prunus armeniaca* synteny and protein identity.

**Gene name**	***Prunus persica***			***Prunus armeniaca***		***Prunus persica vs Prunus armeniaca***		
	**Sequence identifier**	**Gene location**	**LG**	**Obtained sequence**	**Position**	**Synteny block**	**Identity**	**E-value**
PpePAL1	Prupe.2G211800.1	Pp02:24393791-24397929	2	PARG18722	LG5:20435326-20438772	apppB272	710/719 (98.75%)	0
PpePAL2	Prupe.6G235400.1	Pp06:23639324-23642649	6	PARG02214	LG1:17635804-17638656	apppB018	711/717 (99.16%)	0
PpeDFR	Prupe.1G376400.1	Pp01:34110835-34113473	1	PARG07267	LG2: 29288431-29290653	apppB056	333/346 (96.24%)	0
PpeFLS1	Prupe.1G502700.1	Pp01:41577480-41579444	1	PARG08425	LG2:36614060-36616498	apppB057	328/335 (97.91%)	0
PpeFLS2	Prupe.1G502800.1	Pp01:41580633-41583079	1	PARG08426	LG2:36622560-36624610	apppB057	330/338 (97.63%)	0

Table S3. *Arabidopsis thaliana* and *Prunus armeniaca* protein identity.

Arabidopsis thaliana	***Prunus armeniaca***		***Arabidopsis thaliana* vs *Prunus persica***	
	**Gene**	**ID**	**Identity with apricot**	**E-value**
AtPAL1	*ParPAL2*	PARG02214m01	591/705 (83.83%)	0
AtPAL2	*ParPAL1*	PARG18722m01	576/692 (83.24%)	0
AtPAL3	*ParPAL1*	PARG18722m01	522/699 (74.68%)	0
AtPAL4	*ParPAL1*	PARG18722m01	579/709 (81.66%)	0
AtDFR	*ParDFR*	PARG07267m01	241/327 (73.7%)	$1.15 \cdot 10^{-161}$
AtFLS1	*ParFLS1*	PARG08425m01	200/333 (60.06%)	$3.35 \cdot 10^{-126}$
AtFLS2	*ParFLS2*	PARG08426m01	105/230 (45.65%)	$2.38 \cdot 10^{-50}$
AtFLS3	*ParFLS1*	PARG08425m01	158/294 (53.74%)	$3.23 \cdot 10^{-94}$

Table S4. p-distance for PAL (A), DFR (B) and FLS (C)proteins.

**A**	***AtPAl1***	***AtPAL2***	***AtPAL3***	***AtPAL4***	***PpePAL1***	***PpePAL2***	***ParPAL1***	***ParPAL2***	***VvPAL1***	***MdPAL1***	***MdPAL2***	***FvPAL1***
AtPAL1												
AtPAL2	0.09 ± 0.01											
AtPAL3	0.26 ± 0.02	0.26 ± 0.02										
ATPAL4	0.19 ± 0.01	0.19 ± 0.01	0.16 ± 0.01									
PpePAL1	0.19 ± 0.01	0.19 ± 0.01	0.24 ± 0.02	0.17 ± 0.01								
PpePAL2	0.18 ± 0.01	0.19 ± 0.01	0.26 ± 0.02	0.20 ± 0.02	0.16 ± 0.01							
ParPAL1	0.19 ± 0.01	0.19 ± 0.01	0.24 ± 0.02	0.18 ± 0.01	0.01 ± 0.00	0.16 ± 0.01						
ParPAL2	0.18 ± 0.01	0.19 ± 0.01	0.26 ± 0.02	0.20 ± 0.02	0.16 ± 0.01	0.01 ± 0.00	0.16 ± 0.01					
VvPAL1	0.23 ± 0.02	0.21 ± 0.02	0.26 ± 0.02	0.19 ± 0.02	0.17 ± 0.02	0.18 ± 0.02	0.17 ± 0.02	0.18 ± 0.02				
MdPAL1	0.20 ± 0.01	0.19 ± 0.01	0.26 ± 0.02	0.19 ± 0.01	0.08 ± 0.01	0.16 ± 0.01	0.09 ± 0.01	0.16 ± 0.01	0.18 ± 0.02			
MdPAL2	0.18 ± 0.01	0.19 ± 0.01	0.26 ± 0.02	0.19 ± 0.01	0.15 ± 0.01	0.07 ± 0.01	0.16 ± 0.01	0.07 ± 0.01	0.18 ± 0.02	0.16 ± 0.01		
FvPAL1	0.22 ± 0.02	0.22 ± 0.02	0.26 ± 0.02	0.20 ± 0.01	0.16 ± 0.01	0.19 ± 0.01	0.16 ± 0.01	0.19 ± 0.01	0.21 ± 0.02	0.15 ± 0.01	0.19 ± 0.01	
FvPAL2	0.20 ± 0.01	0.21 ± 0.01	0.27 ± 0.02	0.20 ± 0.02	0.17 ± 0.01	0.11 ± 0.01	0.17 ± 0.01	0.11 ± 0.01	0.19 ± 0.02	0.17 ± 0.01	0.10 ± 0.01	0.19 ± 0.01

**B**	***AtDFR***	***PpeDFR***	***ParDFR***	***VvDFR***	***FvDFR***
AtDFR					
PpeDFR	0.31 ± 0.02				
ParDFR	0.30 ± 0.02	0.04 ± 0.01			
VvDFR	0.42 ± 0.04	0.39 ± 0.04	0.39 ± 0.04		
FvDFR	0.29 ± 0.02	0.17 ± 0.02	0.17 ± 0.02	0.41 ± 0.04	
MdDFR	0.36 ± 0.05	0.24 ± 0.04	0.24 ± 0.04	0.60 ± 0.05	0.31 ± 0.05

**C**	***ATFLS1***	***AtFLS2***	***AtFLS3***	***AtFLS3***	***PpeFLS1***	***PpeFLS2***	***ParFLS1***	***ParFLS2***	***VvFLS1***	***MdFLS1***
AtFLS1										
AtFLS2	0.39 ± 0.03									
AtFLS3	0.30 ± 0.03	0.40 ± 0.03								
PpeFLS1	0.42 ± 0.03	0.57 ± 0.03	0.48 ± 0.03							
PpeFLS2	0.42 ± 0.03	0.57 ± 0.03	0.48 ± 0.03	0.22 ± 0.02						
ParFLS1	0.43 ± 0.03	0.57 ± 0.03	0.48 ± 0.03	0.02 ± 0.01	0.22 ± 0.02					
ParFLS2	0.42 ± 0.03	0.57 ± 0.03	0.49 ± 0.03	0.22 ± 0.02	0.02 ± 0.01	0.23 ± 0.02				
VvFLS1	0.37 ± 0.03	0.56 ± 0.04	0.50 ± 0.03	0.29 ± 0.03	0.28 ± 0.03	0.30 ± 0.03	0.28 ± 0.03			
FvFLS1	0.43 ± 0.03	0.57 ± 0.03	0.49 ± 0.03	0.22 ± 0.02	0.20 ± 0.02	0.22 ± 0.02	0.20 ± 0.02	0.28 ± 0.03		
MdFLS1	0.44 ± 0.04	0.58 ± 0.04	0.50 ± 0.04	0.23 ± 0.03	0.33 ± 0.04	0.23 ± 0.03	0.33 ± 0.04	0.35 ± 0.05	0.27 ± 0.04	
MdFLS2	0.60 ± 0.03	0.70 ± 0.03	0.65 ± 0.03	0.52 ± 0.03	0.45 ± 0.03	0.52 ± 0.03	0.46 ± 0.03	0.66 ± 0.04	0.52 ± 0.03	0.32 ± 0.04

Table S5. Similarity (1-p-distance) among protein sequences of PAL (A), DFR (B), FLS (C).

**A**	***AtPAl1***	***AtPAL2***	***AtPAL3***	***AtPAL4***	***PpePAL1***	***PpePAL2***	***ParPAL1***	***ParPAL2***	***VvPAL1***	***MdPAL1***	***MdPAL2***	***FvPAL1***
AtPAL1												
AtPAL2	0.91 ± 0.01											
AtPAL3	0.74 ± 0.02	0.74 ± 0.02										
ATPAL4	0.81 ± 0.01	0.81 ± 0.01	0.84 ± 0.01									
PpePAL1	0.81 ± 0.01	0.81 ± 0.01	0.76 ± 0.02	0.83 ± 0.01								
PpePAL2	0.82 ± 0.01	0.81 ± 0.01	0.74 ± 0.02	0.80 ± 0.02	0.84 ± 0.01							
ParPAL1	0.81 ± 0.01	0.81 ± 0.01	0.76 ± 0.02	0.82 ± 0.01	0.99 ± 0.00	0.84 ± 0.01						
ParPAL2	0.82 ± 0.01	0.81 ± 0.01	0.74 ± 0.02	0.80 ± 0.02	0.84 ± 0.01	0.99 ± 0.00	0.84 ± 0.01					
VvPAL1	0.77 ± 0.02	0.79 ± 0.02	0.74 ± 0.02	0.81 ± 0.02	0.83 ± 0.02	0.82 ± 0.02	0.83 ± 0.02	0.82 ± 0.02				
MdPAL1	0.80 ± 0.01	0.81 ± 0.01	0.74 ± 0.02	0.81 ± 0.01	0.92 ± 0.01	0.84 ± 0.01	0.91 ± 0.01	0.84 ± 0.01	0.82 ± 0.02			
MdPAL2	0.82 ± 0.01	0.81 ± 0.01	0.74 ± 0.02	0.81 ± 0.01	0.85 ± 0.01	0.93 ± 0.01	0.84 ± 0.01	0.93 ± 0.01	0.82 ± 0.02	0.84 ± 0.01		
FvPAL1	0.78 ± 0.02	0.78 ± 0.02	0.74 ± 0.02	0.80 ± 0.01	0.84 ± 0.01	0.81 ± 0.01	0.84 ± 0.01	0.81 ± 0.01	0.79 ± 0.02	0.85 ± 0.01	0.81 ± 0.01	
FvPAL2	0.80 ± 0.01	0.79 ± 0.01	0.73 ± 0.02	0.80 ± 0.02	0.83 ± 0.01	0.89 ± 0.01	0.83 ± 0.01	0.89 ± 0.01	0.81 ± 0.02	0.83 ± 0.01	0.90 ± 0.01	0.81 ± 0.01

**B**	***AtDFR***	***PpeDFR***	***ParDFR***	***VvDFR***	***FvDFR***
AtDFR					
PpeDFR	0.69 ± 0.02				
ParDFR	0.70 ± 0.02	0.96 ± 0.01			
VvDFR	0.58 ± 0.04	0.61 ± 0.04	0.61 ± 0.04		
FvDFR	0.71 ± 0.02	0.83 ± 0.02	0.83 ± 0.02	0.59 ± 0.04	
MdDFR	0.64 ± 0.05	0.76 ± 0.04	0.76 ± 0.04	0.40 ± 0.05	0.69 ± 0.05

C	*ATFLS1*	*AtFLS2*	*AtFLS3*	*AtFLS3*	*PpeFLS1*	*PpeFLS2*	*ParFLS1*	*ParFLS2*	*VvFLS1*	*MdFLS1*
AtFLS1										
AtFLS2	0.61 ± 0.03									
AtFLS3	0.70 ± 0.03	0.60 ± 0.03								
PpeFLS1	0.58 ± 0.03	0.43 ± 0.03	0.52 ± 0.03							
PpeFLS2	0.58 ± 0.03	0.43 ± 0.03	0.52 ± 0.03	0.78 ± 0.02						
ParFLS1	0.57 ± 0.03	0.43 ± 0.03	0.52 ± 0.03	0.98 ± 0.01	0.78 ± 0.02					
ParFLS2	0.58 ± 0.03	0.43 ± 0.03	0.51 ± 0.03	0.78 ± 0.02	0.98 ± 0.01	0.77 ± 0.02				
VvFLS1	0.63 ± 0.03	0.44 ± 0.04	0.50 ± 0.03	0.71 ± 0.03	0.72 ± 0.03	0.70 ± 0.03	0.72 ± 0.03			
FvFLS1	0.57 ± 0.03	0.43 ± 0.03	0.51 ± 0.03	0.78 ± 0.02	0.80 ± 0.02	0.78 ± 0.02	0.80 ± 0.02	0.72 ± 0.03		
MdFLS1	0.56 ± 0.04	0.42 ± 0.04	0.50 ± 0.04	0.77 ± 0.03	0.67 ± 0.04	0.77 ± 0.03	0.67 ± 0.04	0.65 ± 0.05	0.73 ± 0.04	
MdFLS2	0.40 ± 0.03	0.30 ± 0.03	0.35 ± 0.03	0.48 ± 0.03	0.55 ± 0.03	0.48 ± 0.03	0.54 ± 0.03	0.34 ± 0.04	0.48 ± 0.03	0.68 ± 0.04

Table S6: Genetic expression of studied genotypes. Different letter means significant differences among genotypes.

Genotype	2019														
	ParDFR			***ParFLS1***			***ParFLS2***			***ParPAL1***			***ParPAL2***		
	**Mean**	**Sd**	**Sig.**	**Mean**	**Sd**	**Sig.**	**Mean**	**Sd**	**Sig.**	**Mean**	**Sd**	**Sig.**	**Mean**	**Sd**	**Sig.**
**Canino**	0.75	0.15	bc	0.97	0.48	a	2.34	0.39	ab	1.18	0.06	ab	1.77	0.29	bc
**Dama Rosa**	0.38	0.04	ab	0.61	0.32	a	2.19	0.58	ab	1.13	0.17	ab	0.56	0.02	ab
**Dama Taronja**	2.52	0.25	d	0.51	0.35	a	0.42	0.14	ab	1.95	0.14	b	1.78	0.33	bc
**GG9310**	0.50	0.30	ab	0.84	0.50	a	5.05	2.17	c	1.00	0.52	ab	0.46	0.20	a
**GG979**	0.12	0.03	a	3.73	2.56	a	0.84	0.10	ab	1.68	0.59	b	0.29	0.10	a
**Goldrich**	0.51	0.05	ab	1.07	0.41	a	0.24	0.08	a	1.94	0.60	b	0.86	0.30	ab
**GP9817**	0.38	0.07	ab	1.21	1.22	a	1.25	0.04	ab	1.17	0.20	ab	0.63	0.10	ab
**HG9821**	0.93	0.02	bc	0.41	0.25	a	2.59	0.76	b	1.13	0.32	ab	1.06	0.10	abc
**HG9850**	2.27	0.13	d	0.34	0.24	a	0.76	0.06	ab	1.01	0.21	ab	2.29	0.70	cd
**HM964**	1.10	0.15	c	0.76	0.30	a	1.44	0.09	ab	2.02	0.29	b	1.06	0.37	abc
**Mitger**	2.27	0.36	d	0.57	0.01	a	0.81	0.19	ab	1.65	0.54	b	3.44	0.97	d
**SEOP934**	0.83	0.18	bc	1.69	1.63	a	1.02	0.28	ab	0.30	0.02	a	0.78	0.05	ab

Genotype	2020														
	ParDFR			***ParFLS1***			***ParFLS2***			***ParPAL1***			***ParPAL2***		
	**Mean**	**Sd**	**Sig.**	**Mean**	**Sd**	**Sig.**	**Mean**	**Sd**	**Sig.**	**Mean**	**Sd**	**Sig.**	**Mean**	**Sd**	**Sig.**
**Canino**	0.57	0.16	a	4.09	3.10	b	0.54	0.08	ab	3.74	1.42	b	1.95	0.58	bc
**Dama Rosa**	0.44	0.14	a	2.91	0.81	ab	1.21	0.37	bc	1.10	0.14	a	0.90	0.37	abc
**Dama Taronja**	0.27	0.05	a	0.54	0.32	a	0.13	0.02	a	0.41	0.04	a	0.81	0.10	abc
**GG9310**	0.17	0.03	a	2.20	1.79	ab	1.18	0.34	bc	0.70	0.22	a	0.17	0.01	ab
**GG979**	0.14	0.03	a	0.41	0.20	a	0.57	0.17	ab	1.64	0.44	a	0.49	0.12	abc
**Goldrich**	0.97	0.33	ab	0.30	0.13	a	0.04	0.02	a	3.58	1.14	b	2.00	0.86	c
**GP9817**	0.16	0.05	a	0.41	0.29	a	0.97	0.03	bc	0.32	0.01	a	0.16	0.04	a
**HG9821**	2.05	0.53	bc	2.54	1.39	ab	1.14	0.29	bc	1.33	0.32	a	0.74	0.17	abc
**HG9850**	2.66	0.97	c	1.95	1.46	ab	0.64	0.07	abc	1.01	0.36	a	4.55	1.48	d
**HM964**	0.61	0.11	a	1.98	0.70	ab	1.38	0.30	c	0.38	0.03	a	0.37	0.06	abc
**Mitger**	5.14	0.14	d	0.59	0.31	a	3.34	0.53	d	0.59	0.14	a	4.68	0.57	d
**SEOP934**	1.11	0.13	ab	1.19	0.56	a	2.61	0.09	d	0.48	0.05	a	1.15	0.24	abc

Table S7: Linear regression model in caffeate-derivates.

NEOCHLORGENIC ACID

2019

Parameter	Estimation	Sd	T	P-value	R2
**CONSTANT**	185.653	42.8202	4.33564	0.0002	
ParDFR	-81.7058	33.5748	-2.43355	0.0216	
ParFLS2	34.5919	10.6549	3.24656	0.003	
ParPAL1	106.167	33.3428	3.1841	0.0035	0.6191
ParPAL2	-108.959	36.882	-2.95426	0.0063	
ParPAL1Par / FLS2	-49.3894	19.9979	-2.46973	0.0199	
ParPAL2 / ParFLS2	92.9533	37.829	2.4572	0.0205	

2020

Parameter	Estimation	Sd	T	P-value	R2
**CONSTANT**	275.686	32.8475	8.39292	0	**0.**0784
ParFLS2	37.5785	22.0911	1.70107	0.0981	

2019-2020

Parameter	Estimation	Sd	T	P-value	R2
**CONSTANT**	294.892	21.027	14.0244	0	
ParPAL2 / ParFLS1	-18.5607	8.70188	-2.13296	0.0366	0.063

CHLOROGENIC ACID

2019

Parameter	Estimation	Sd	T	P-value	R2
**CONSTANT**	290.902	29.6794	9.80146	0	0.293
ParDFR	-84.0371	22.3848	-3.75421	0.0007	

2020

Parameter	Estimation	Sd	T	P-value	R2
**CONSTANT**	386.407	39.5198	9.77757	0	0.04616
ParPAL1	-28.6776	22.355	-1.28283	0.2082	

2019-2020

Parameter	Estimation	Sd	T	P-value	R2
**CONSTANT**	318.278	27.1345	11.7296	0	0.0816
ParPAL2 / ParFLS1	-27.4045	11.2294	-2.44043	0.0173	

CHLOROGENIC AND NEOCHLOROGENIC TOTAL CONTENT

2019

Parameter	Estimation	Sd	T	P-value	R2
**CONSTANT**	525.668	80.5319	6.52744	0	
ParDFR	-241.336	72.6906	-3.32005	0.0024	
ParPAL1	233.953	71.6001	3.26749	0.0028	0.5339
ParPAL2	-204.74	79.8529	-2.56397	0.0158	
ParPAL1 / ParFLS2	-112.661	43.0388	-2.61765	0.0139	
ParPAL2 / ParFLS2	190.943	81.5529	2.34134	0.0263	

2020

Parameter	Estimation	Sd	T	P-value	R2
**CONSTANT**	746.592	62.4755	11.9501	0	0.0810975
ParPAL1	-61.218	35.3404	-1.73224	0.0923	

2019-2020

Parameter	Estimation	Sd	T	P-value	R2
**CONSTANT**	613.17	43.8595	13.9803	0	0.0873
ParPAL2/ParFLS1	-45.9653	18.1509	-2.53239	0.0137	

Table S8: Linear regression model in flavonols.

RUTIN

2019

**Parameter**	**Estimation**	**Sd**	**T**	**P-value**	**R2**
**CONSTANT**	562.793	90.0062	6.25283	0	0.366
ParDFR	-294.562	78.952	-3.7309	0.0009	
ParPAL1	251.179	86.265	2.91171	0.007	
ParPAL2	-251.405	86.1836	-2.91709	0.0069	
ParPAL1 / ParPAL2	-58.0325	26.0671	-2.22627	0.0342	
ParPAL1 / ParFLS2	-151.616	44.6125	-3.39851	0.0021	
ParPAL2 / ParFLS2	304.215	84.1984	3.61308	0.0012	

2020

**Parameter**	**Estimation**	**Sd**	**T**	**P-value**	**R2**
**CONSTANT**	211.629	21.3124	9.92987	0	0.515868
ParFLS1 / ParFLS2	9.19147	2.2127	4.15396	0.0003	
ParPAL2	38.4111	8.62697	4.45244	0.0001	
ParPAL1 / ParFLS2	-2.59935	0.720061	-3.6099	0.0011	

2019-2020

**Parameter**	**Estimation**	**Sd**	**T**	**P-value**	**R2**
**CONSTANT**	356.984	23.5464	15.1609	0	0.0425
ParFLS1	-19.4258	11.2636	-1.72466	0.0892	

QUERCETIN_3-GLUCURONIDE

2019

**Parameter**	**Estimation**	**Sd**	**T**	**P-value**	**R2**
**CONSTANT**	48.7709	8.15762	5.97857	0	0.1815
ParPAL2	-23.0258	8.99826	-2.55892	0.0156	
ParPAL1 / ParFLS2	-8.35646	3.44867	-2.42309	0.0214	
ParPAL2 / ParFLS2	17.4134	6.84919	2.5424	0.0162	

2020

**Parameter**	**Estimation**	**Sd**	**T**	**P-value**	**R2**
**CONSTANT**	28.3714	2.98273	9.51191	0	0.5852
ParFLS1 / ParFLS2	1.40385	0.294044	4.7743	0	
ParPAL1 / ParPAL2	-4.4801	1.31522	-3.40636	0.0019	
ParPAL1 / ParFLS2	-0.448444	0.0963081	-4.65634	0.0001	

2019-2020

**Parameter**	**Estimation**	**Sd**	**T**	**P-value**	**R2**
**CONSTANT**	32.7225	3.31507	9.87083	0	0.04786
ParPAL1 / ParPAL2	-2.83612	1.52274	-1.8625	0.0668	

RUTIN+QUERCETIN-3-GLUCURONIDE

2019					
**Parameter**	**Estimation**	**Sd**	**T**	**P-value**	**R2**
CONSTANT	615.21	98.5015	6.24569	0	
ParDFR	-313.956	86.4039	-3.63358	0.0011	
ParPAL1	282.717	94.4072	2.99465	0.0057	
ParPAL2	-293.832	94.3181	-3.11533	0.0042	0.368
ParPAL1 / ParPAL2	-64.1542	28.5275	-2.24885	0.0326	
ParPAL1 / ParFLS2	-172.127	48.8232	-3.52552	0.0015	
ParPAL2 / FLS2	341.894	92.1456	3.71037	0.0009	

2020					
**Parameter**	**Estimation**	**Sd**	**T**	**P-value**	**R2**
CONSTANT	227.956	23.062	9.88451	0	
ParFLS1 /Par FLS2	10.8076	2.39435	4.51382	0.0001	0.536877
ParPAL2	41.3101	9.33517	4.42521	0.0001	
ParPAL1 / ParFLS2	-3.12702	0.779172	-4.01326	0.0004	

2019-2020					
**Parameter**	**Estimation**	**Sd**	**T**	**P-value**	**R2**
CONSTANT	387.332	25.922	14.9423	0	0.0417
ParFLS1	-21.1966	12.3999	-1.70941	0.092	

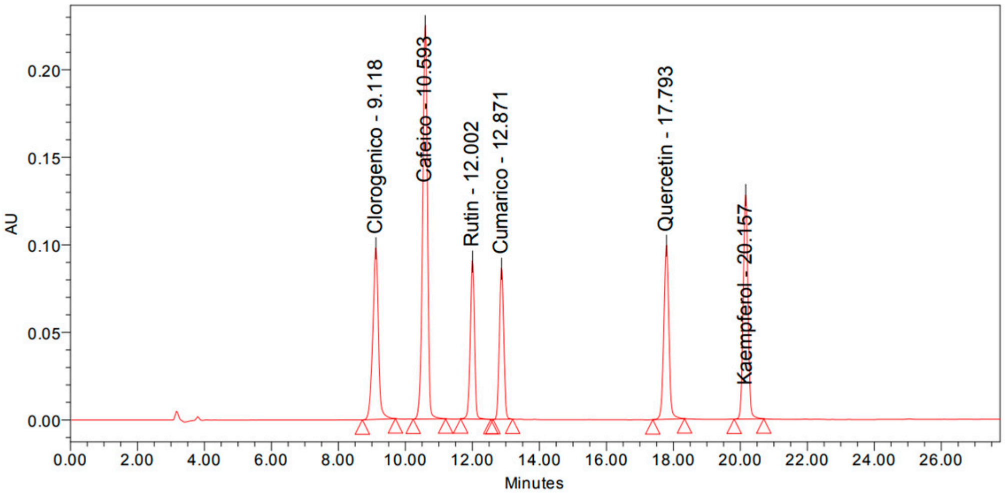

Figure S1. Standard chromatogram and retention times for chlorogenic acid (Clorogenico), caffeic acid (Cafeico), rutin, coumaric (Cumarico), quercetin and kaempferol.

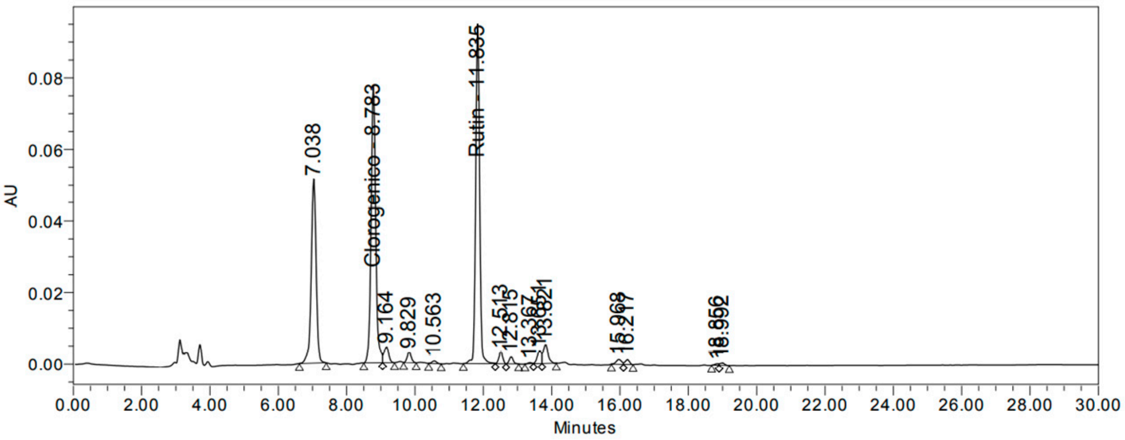

Figure S2. 'Canino' chromatogram from one biological replicate sample.
